# General Aspects of Metal Ions as Signaling Agents in Health and Disease

**DOI:** 10.3390/biom10101417

**Published:** 2020-10-07

**Authors:** Karolina Krzywoszyńska, Danuta Witkowska, Jolanta Świątek-Kozłowska, Agnieszka Szebesczyk, Henryk Kozłowski

**Affiliations:** 1 Institute of Health Sciences, University of Opole, 68 Katowicka St., 45-060 Opole, Poland; jolanta.swiatekkozlowska@uni.opole.pl (J.Ś.-K.); agnieszka.szebesczyk@uni.opole.pl (A.S.); henryk.kozlowski@chem.uni.wroc.pl (H.K.); 2 Faculty of Chemistry, University of Wrocław, 14 F. Joliot-Curie St., 50-383 Wrocław, Poland

**Keywords:** cell signaling, metal homeostasis, ferroptosis

## Abstract

This review focuses on the current knowledge on the involvement of metal ions in signaling processes within the cell, in both physiological and pathological conditions. The first section is devoted to the recent discoveries on magnesium and calcium-dependent signal transduction—the most recognized signaling agents among metals. The following sections then describe signaling pathways where zinc, copper, and iron play a key role. There are many systems in which changes in intra- and extra-cellular zinc and copper concentrations have been linked to important downstream events, especially in nervous signal transduction. Iron signaling is mostly related with its homeostasis. However, it is also involved in a recently discovered type of programmed cell death, ferroptosis. The important differences in metal ion signaling, and its disease-leading alterations, are also discussed.

## 1. Introduction

Signal transduction and spreading is a key cellular process in maintaining life and its development. Typical chemical signaling comprises the release of a transmitter from one cell and its interaction with selected detectors on the surface of another. If the transmitter is taken up into the cell, it may bind to receptors on the inner part of cell membrane and, thus, stimulate it to react in a required manner to this signal. The molecular mechanisms of reaction to the signal are regulated by a strict spatiotemporal dynamic [1]. The signal effectiveness and transduction differ on the extra and intracellular side of the cell. In general, extracellular signaling depends on exposure to a sufficient carrier concentration. Simultaneously, the changes in the timing and frequency of a messenger are crucial for the intracellular signals [2]. Changes in the concentration of metal ions affect the signaling processes in both excitable and non-excitable cells on both sides of the cellular membrane [3,4,5,6].

The major groups of chemical neurotransmitters in excitable cells are amino acids, amines, or neuropeptides. Recent studies also indicate that metal ions, such as zinc and copper, may be released to the synaptic cleft [7,8,9]. This phenomenon is still not fully understood; however, it is a very interesting example of signaling involving metal ions. Many new studies and conclusions have appeared in this field recently and will be discussed in this review. Studies show that both the normal aging of the brain and the development of diseases, such as neurodegenerative and psychiatric disorders, are manifested by deregulation of the management of metal ions such as iron, zinc, and copper. That disproportion can have a direct impact on the neurotransmission coordinated by these ions and cellular processes necessary for proper functioning of nerve cells [10,11]. Furthermore, iron ions, along with reactive oxygen species (ROS), were connected with a recently discovered form of programmed cell death named ferroptosis [12]. While the pathological role of metal ions is still under investigation, it actually may arise from homeostasis disorders, which, in turn, may be related to disorders of metal ions sensing by different cells.

## 2. Magnesium

Magnesium (Mg) is an essential element that acts as a cofactor in many enzymes involved in the synthesis, folding, and stability of small and large biomolecules [13]. This most abundant free divalent cation in a cell, is one of the essential macronutrients in organism growth and development. Until now, more than 600 enzymatic reactions involving magnesium have been discovered [14]. About 99% of total body magnesium is distributed between different tissues in humans, amounting to approximately 25 g of magnesium in total for adults, with the largest proportion found in bones [15]; only 1–2% of the total magnesium is located in the blood. Mg^2+^ can act as an antagonist to reduce Ca^2+^ signaling in endothelium [16]. In spite of obvious chemical similarities between calcium and magnesium, major differences often prevail. For instance, the hydrated magnesium cation is hard to dehydrate, making it almost impossible for it to pass through narrow channels in biological membranes, which are no obstacles for calcium move [17]. Mg^2+^ binds water molecules more tightly than other cations, and for this reason the energy required for its transport is several times greater than that required for the transport of other cations [18]. Magnesium exhibits unique characteristics- the largest hydrated radius (0.428 nm) and the smallest ionic radius (0.072 nm) [19].

Mg^2+^ homeostasis is achieved through a balance of its uptake, intracellular storage, and efflux. Its deficiency can have destructive effects on the life of the cell [20]. Indeed, disorders of Mg^2+^ homeostasis are involved in neurodegenerative and cardiovascular diseases, bone disorders, asthma, cancer and diabetes [14,20,21,22]. It seems that magnesium deficiency can play an important role in the induction of inflammation in some of the mentioned pathologic conditions [21].

**Figure 1:**
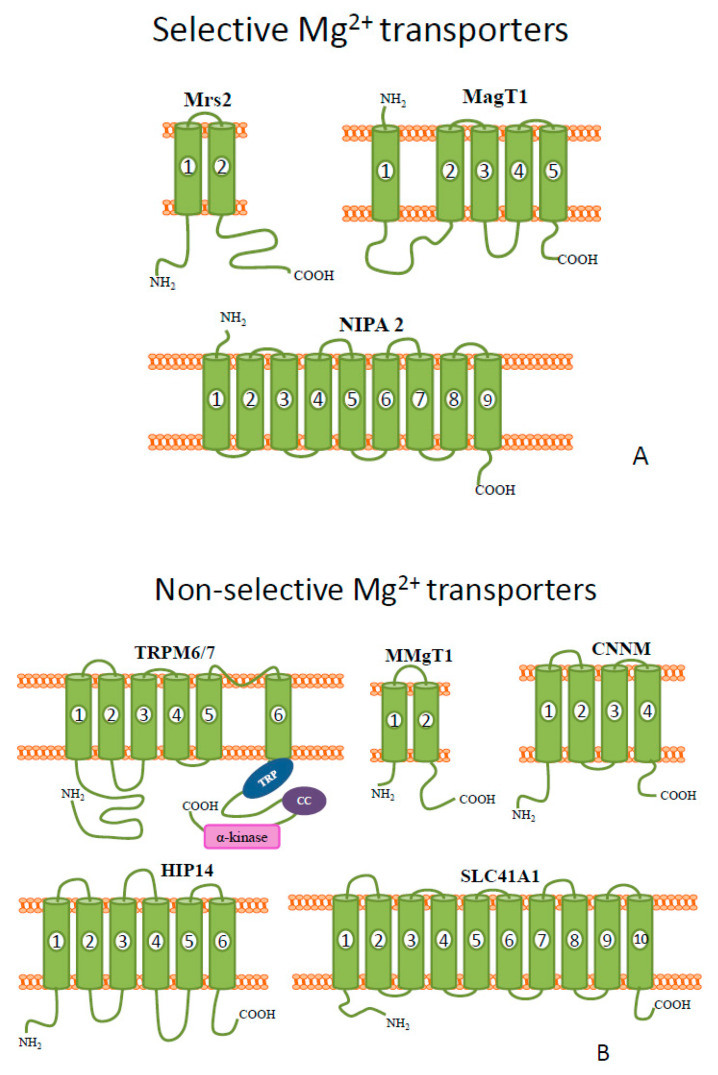
Schematic diagrams of the representative members of mammalian (**A**) selective and (**B**) non-selective Mg^2+^ transporters.

Its cellular homeostasis in vertebrates is regulated by the combined action of mitochondrial RNA splicing 2 (Mrs2), transient receptor potential melastatin 6/7(TRPM6/7), solute carrier family 41 (SLC41), membrane Mg^2+^ transporter 1 (MagT1), non-imprinted in Prader-Willi/Angelman syndrome protein (NIPA), membrane Mg^2+^ transporters (MMgTs), cyclin and cystathionine β-synthase domain magnesium transport mediators (CNNMs), and huntingtin-interacting protein 14 (HIP14) transporters [14,23,24]. The majority of proteins belonging to these families also transport other divalent cations across membranes (Figure 1B); only some of them (Figure 1A) are selective for Mg^2+^ ions [24]. Nonetheless, very recent data show that extracellular Mg^2+^ ions enter some tissues mainly through the TRPM7 channel and MagT1 transporter [16,25]. There are no evident uniform amino acid sequence similarities among the various magnesium transporters, even between MagT1 and NIPA2.

Mrs2 was the first mammalian magnesium transporter identified at the molecular level [26]. Using single channel patch clamping, Schindl et al. have shown that Mrs2 forms a Mg^2+^ selective channel of high conductance (155pS) [27]. Moreover, this channel is also permeable for Ni^2+^, with a lower conductance, and there was no permeability for Ca^2+^, Mn^2+^, and Co^2+^ ions [27].

MMgT1 and MMgT2 (membrane Mg^2+^ transporter 1 and 2) belong to a novel family of magnesium transporters with no known similarities to other transporters [24]. They were identified by differential gene expression using microarray analysis by Goytain and Quamme [28]. It was shown that MMgT1 and MMgT2 proteins reside in the Golgi and post-Golgi vesicles and both, as determined by two-electrode voltage-clamp analysis and fluorescence measurements, mediate magnesium ions [28].

Another protein responsible for magnesium homeostasis—magnesium transporter 1 (MagT1) is a plasma membrane Mg^2+^ transporter, highly-conserved across different eukaryotic species [29].

The full-length protein is composed of 367 amino acids with a large N-terminal segment, four transmembrane domains (TMs), and a small C-terminal tail (Figure 1A); it shows no structural similarity to any other magnesium transporters [30]. Transport of Mg^2+^ by this protein is rheogenic and voltage-dependent. It is highly selective for magnesium ions, as shown by fluorescence and voltage-clamp methods [24]. Recently, it has been demonstrated that humans lacking functional MagT1 have a selective deficiency in both immune and nonimmune glycoproteins [29]. Studies involving patients who had suffered from X-link immunodeficiency with magnesium defect and Epstein- Barr virus infection and neoplasia (XMEN) disease revealed that MagT1 serves as a kinetic regulator of signaling in lymphocyte T cells but not in B cells. It was suggested that Mg^2+^ influx may promote rapid spatial integration of antigen and costimulatory receptor signals critical for T cell activation. Moreover, MagT1 deficiency impairs T-cell receptor induced Mg^2+^ and Ca^2+^ influxes [31]. On the other hand, studies on mice with magT1 genetic deletion proved MagT1 is not required for lymphocyte homeostasis. MagT1-deficient cluster of differentiation 4^+^ (CD4^+^) proliferation was normal in multiple in vitro proliferation assays [32]. This discrepancy could arise due to differences in human and mouse physiology. It could be also that MagT1 has other functions that, when distorted, contribute to immunodeficiency [33].

The second highly Mg^2+^ selective transporter, non-imprinted in Prader-Willi/Angelman syndrome protein 2 (NIPA2) was identified using microarray analysis [34].

Other members of NIPA family belong to nonselective magnesium transporters. NIPA2 consists of 360 amino acids and has nine transmembrane protein domains (Figure 1A); it is located in many tissues, but particularly plentiful in renal cells [34]. It was suggested that *NIPA2* mutations may contribute to childhood absence epilepsy, as mutant proteins were accumulated in the cytoplasm, which reduced intracellular Mg^2+^concentration in the neurons and affected neuronal excitability [35]. This hypothesis is supported by the results of a recent study, where the dysfunction of NIPA2 proved to reduce big potassium (BK) channel currents. Furthermore, it was shown that the decreased currents of BK channels enhanced neuronal excitability [36]. NIPA2 is also associated with type 2 diabetes [37]. This highly-selective magnesium ion transporter was shown to regulate osteoblast apoptosis by affecting the intracellular magnesium level and further affecting the osteogenic capacity of osteoblasts. These results suggest that NIPA2 could be a potential target for the treatment of type 2 diabetes osteoporosis [37].

CNNMs have been shown to be encoded by *Acdp* genes [24]. Some researchers suggest that CNNMs serve as direct transporters that extrude Mg^2+^ ions from the cell by exchanging it with Na^+^ ions [38,39]. On the other hand, there is an evidence suggesting that they can act either as intracellular Mg^2+^ sensors or as Mg^2+^ homeostatic mediators [40,41,42]. Nevertheless, several structural characteristics support their direct involvement in the Mg^2+^ extrusion [15].

Nowadays, TRPM7 and its homologue—TRPM6 seem to be the most investigated Mg^2+^ transporters. The significance of TRPM7 in cellular magnesium regulation has been analyzed in many cell types, including cardiomyocytes, osteoblasts, tumor cells and leukocytes, to name a few [43,44,45]. In vascular cells, that transporter occurs to be the central cation channel involved in controlling [Mg^2+^]. Montezano and coworkers have shown that magnesium prevents vascular calcification and osteogenic differentiation by restoring TRPM7 activity, counteracting calcium actions, and increasing expression of anticalcification proteins [46].

Both TRPM6 and TRPM7 proteins comprise 6 TMs and a channel pore, permeable also for Ca^2+^, Mn^2+^, Co^2+^ and Zn^2+^ ions [47], located between segments 5 and 6 (Figure 1B). In the plasma membrane, TRPM7 functions as a homodimer, although it can also heterodimerize with its analogue TRPM6. TRPM6 is mostly expressed in intestines and kidneys and TRPM7 is ubiquitously expressed [25,48]. The role of TRPM7 in Mg^2+^ homeostasis has been questioned by Jin et al., since the deletion of this transporter did not affect the maintenance of total cellular magnesium ions level [49]. Nonetheless, as stated above, Mg^2+^ is typically regulated by MagT1 in immune cells. In DT40 cells and colon carcinoma cells, the lack of TRPM7 was associated with increased expression of MagT1. This result implies that the discrepancies may come from different cell types studied [50,51].

Emerging evidence demonstrates a crucial role for TRPM6 and TRPM7 chanzymes (protein that shows fused channel and enzyme activities) in growth factor signaling through receptor tyrosine kinases (RTKs) [25]. RTKs, typically activated by growth factors, are membrane-associated receptors [52]. They induce phosphorylation and activation of intracellular non-receptor kinases which can trigger critical signaling pathways and cell functions, such as migration, contraction, proliferation, and differentiation [25].

Magnesium ions are important in the regulation of kinase activity. RTKs influence TRPM7, which in turn can influence tyrosine kinase signaling. Humans have around 60 known RTKs, which fall into 20 subfamilies [53]; however, the C-terminal kinase ofTRPM7 (alpha-kinase) displays little amino acid sequence similarity to other known kinases [45,54]. Recently, studies showing a significant role for TRPM7’s kinase in regulating proteasome-mediated turnover of the channel and controlling its cellular localization in polarized epithelial cells, have been conducted [55]. The phosphorylation of Ser-1360 has been shown to be critical for controlling protein stability and cellular localization of the channel [55]. The intrinsic kinase activity of TRPM7 constitutes a mechanism that allows the channel to respond rapidly to different cellular conditions and requirements.

Next magnesium transporter—HIP14 acts as a chanzyme as well. Goytain and Quamme demonstrated, by fluorescence and voltage-clamp techniques, that HIP14 mediates Mg^2+^ flux [30]. That work revealed that HIP14 trafficking from Golgi to post-Golgi vesicles increases when extracellular levels of magnesium ions are lowered [30]. Moreover, HIP14 contain 11 cysteine residues that might function as palmitoylation sites, what in turn can influence HIP14-mediated magnesium transport [24]. Palmitoylation (reversible, posttranslational covalent attachment of palmitic acid to cysteine residues) increases the hydrophobicity of proteins and thereby regulates their membrane association and subcellular localization [56]. It activates many cation transporters, such as Na^+^, K^+^, and Ca^2+^ channels [57,58]. Singaraja et al. revealed that altered palmitoylation of HIP14 substrates could contribute to the pathogenesis of Huntington disease (HD) [59]. Patients with HD often demonstrate abnormalities of iron homeostasis as well, what shows that huntingtin-HIP14 interactions are more complex.

Solute carrier family 41 member 1 (SLC41A1) protein was also shown to be differentially regulated by Mg^2+^ ions [60]. It is composed of 10 TMs (Figure 1B). SLC41A1 protein has been determined to transport, apart from magnesium ions, many other divalent cations. The transport of Mg^2+^by SLC41A1 had been previously shown to be rheogenic and voltage dependent, but not coupled to Na^+^ or Cl^−^ ions [60]. More recent data suggest that SLC41A1 is an NME (Na^+^/Mg^2+^ exchanger) and the major cellular Mg^2+^ efflux system [61]. Under acute oxidative stress, SLC41A1 together with two other proteins might act as molecular mechanisms causing significant Mg^2+^ wasting. That situation can result in decreased cellular metabolism (mitochondrial dysfunction) and pro-apoptotic responses [20]. In 2017, an interesting study was carried out in humans regarding the efficacy of magnesium supplementation on the transcription of TRPM6/7 and SLC41A1 transporters [62]; the results showed a notable increase only in the expression of TRPM6. However, that study had several limitations, the most prominent of which being the fact that intracellular levels of magnesium were not measured [62].

A very interesting study on the effects of magnesium signaling on the structural and functional development of neuronal cells has been carried out by Yamanaka et al. [63]. It was shown that the activation of gamma-aminobutyric acid A (GABA_A_) receptors mediates the GABA-induced cytosolic [Mg]^2+^ increase in immature neurons independently of calcium signals. Moreover, cytosolic magnesium regulates the signaling activities of extracellular signal-regulated kinase (ERK) negatively, cyclic AMP response element binding protein (CREB) positively, and mammalian target of rapamycin (mTOR) sigmoidally [63]. As these intracellular signaling pathways regulate neuronal growth and differentiation, magnesium, by activating CREB and mTOR signaling, enhances the maturation of neural networks [64].

As a result of the contradictory observations regarding magnesium, its regulatory system and the roles of intracellular Mg^2+^ are controversial [64].

It is worth to mention that there are also unique prokaryotic Mg^2+^ transport systems with unusual mechanisms for mediating Mg^2+^ movement through the membrane [65]. However, they are beyond the scope of this review. For more information on magnesium transporters and signaling please see these excellent recent reviews [14,24,25,40].

## 3. Calcium

Calcium ions (Ca^2+^) are prevalent second messengers that regulate physiological cell functions in almost all living beings. The right level of calcium in the cytoplasm is maintained by calcium-permeable channels, transporters, and ATPases. Only 0.1% of calcium is actually present in extracellular fluid, where calcium exists in different fractions, such as protein-bound calcium (40%), free or ionized (48%), and complexed to other inorganic compounds (12%) [66]. When the cell is under resting conditions, the cytosolic free calcium concentration [Ca^2+^] is maintained at approximately 100 nM. More calcium is stored in some organelles, such as the Golgi apparatus and endoplasmic reticulum (ER), amounting to hundreds of microM [67]. A close relationship exists between the ER and endosomal system to initiate calcium signaling, or to store and buffer the Ca^2+^ after its release [68]. There are two pathways of increasing [Ca^2+^]_i_ which coexist in cells: the release from intracellular stores, mainly ER, or the influx from the extracellular medium through the opening of calcium-permeable channels and Ca^2+^ transporters located in plasma membrane [69].

**Figure 2:**
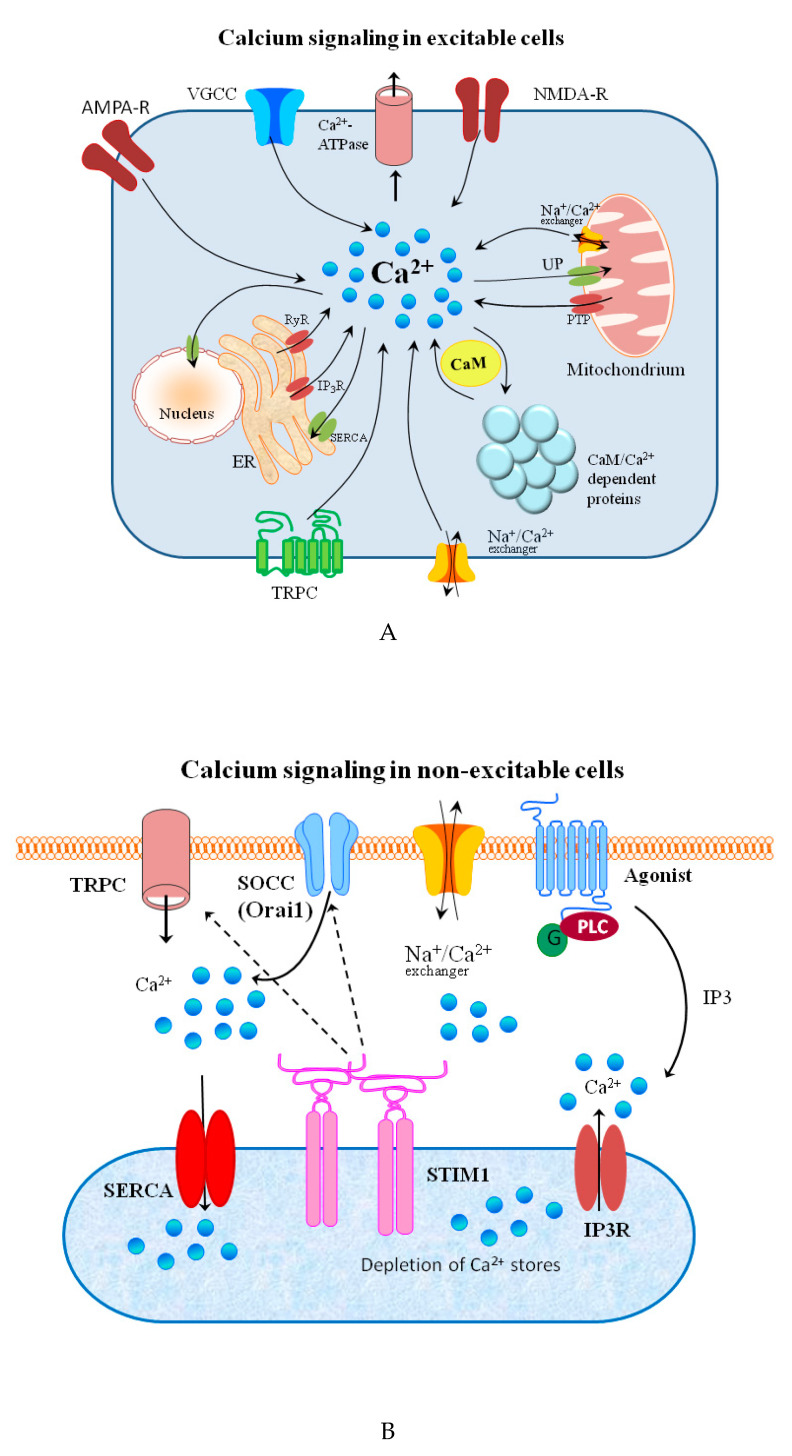
(**A**) Schematic representation of calcium signaling in excitable cells. Voltage-gated calcium channels (VGCC) are found in the membrane of excitable cells. Other sources of calcium influx are calcium-permeable α-amino-3-hydroxy-5-methyl-4-isoxazolepropionic acid (AMPA) and N-methyl-D-aspartate (NMDA) glutamate-type receptors, and transient receptor potential type C (TRPC) channels. cAMP-dependent protein kinases and the Ca^2+^calmodulin-dependent proteins play crucial roles in local signaling and synaptic plasticity. Calcium release from internal stores is mediated by inositol trisphosphate receptors (IP_3_R), ryanodine receptors (RyR), and mitochondrial permeability transition pore (PTP). Ca^2+^ efflux is mediated by the plasma membrane calcium ATPase, the Na^2+^/Ca^2+^ exchanger, and the sarco-/endoplasmic reticulum calcium ATPase (SERCA) and the mitochondrial uniporter (UP). (**B**) Schematic representation of calcium signaling in non-excitable cells. Following the stimulation of G protein-coupled receptors, IP3 produced by phospholipase C (PLC) activates the IP3 receptor (IP3R) and triggers the depletion of ER calcium stores. The stromal interaction molecule 1(STIM1), representing Ca^2+^ sensor, and Orai, belonging to the group of store-operated calcium channels (SOCC) builds the ion-conducting transmembrane protein complex. STIM1 interacts with Orai1 to trigger SOCC opening. Then, SERCA pumps Ca^2+^ back into the ER to refill the stores with Ca^2+^. Transient receptor potential channels (TRPC) can be also activated by phospholipase C stimulation.

The release from internal stores goes through a variety of messengers such as inositol-1,4,5-trisphosphate (IP3), cyclic ADP-ribose (cADPR), nicotinic acid adenine dinucleotide phosphate (NAADP) and others [67,70]. In many electrically non-excitable cells, both processes are coupled in a process that is known as the store-operated calcium entry (SOCE). This mechanism has two important functions in the cell: cellular signaling and store refilling [71]. The influx of Ca^2+^ ions is also regulated by the TRPM chanzymes, by their effects on other channels such as ligand- or voltage-gated Ca^2+^ channels, through modulation of the membrane potential [72]. There is some evidence showing a relationship between TRPM channels, SOCE and the ER store content [71,73]. Two kinds of Ca^2+^ entry channels can be found in cells: voltage-gated calcium channels (VGCCs), which are dominant in excitable cells, and non-voltage-gated channels, which are dominant in non-excitable cells [73]. Recent advances in the structural biology of voltage-gated sodium and calcium channels are reviewed by Catterall and coworkers [74]. Very recently, an interesting study on the influence of Ca^2+^ concentration on voltage-dependent L-type calcium channels’ in fish was published [75]. In another newly published paper, the authors review the interplay between three potassium channels and calcium ions [76]. The calcium ions are pumped out of the cytoplasm by the membrane Ca^2+^-ATPase and the sarcoplasmic/endoplasmic reticulum Ca^2+^-ATPase [77,78]. Schematic representations of calcium-permeable channels, transporters, and ATPases involved in calcium signaling in excitable and non-excitable cells are shown in Figure 2A,B, respectively.

Calcium ions are involved in controlling cell proliferation, differentiation, secretion, maturation, mobility, and contraction [53,69,79]. Environmental stimuli mobilize intrinsic calcium stores, and selectively regulate its function alone or employing primary cell function [80].

Functional abnormalities in proteins that mediate Ca^2+^ transport and homeostasis usually lead to a wide range of diseases and pathogenic states, including cancer, heart failure, diabetes, and neurodegenerative disease [73]. Moreover, Ca^2+^ plays numerous roles in the immune system; calcium signaling is essential for T cell activation, tolerance of self-antigens, differentiation, and development [81]. The key regulators of T cells are the nuclear factor of activated T cells (NFAT) proteins [82]. It has been shown recently that suppression of Ca^2+^-NFAT signaling weakens T cell activation, clonal expansion, and infection clearance in fish [83].

There are dendritic cells (DC) in the mammalian immune system, also known as accessory cells, whose role is to capture and process antigens, then present antigens on the cell surface to the T cells. An increase in the [Ca^2+^]_i_ acts as a signal that influences a broad range of dendritic cell (DC) functions [84]. The initiation and maintenance of these functions is induced by antigen receptor engagement. It has been shown that Ca^2+^ ions activate nonselective cation channel TRPM4 in DC cells. Authors conclude that TRPM4-regulated calcium homeostasis has been important for DC mobility, but not its maturation [74,85].

Ca^2+^ signaling pathways have a crucial role in signaling in excitable cells [86]. The role of Ca^2+^ in neuronal cells is composed of synaptic transmission and modulation of many signaling cascades upon activation of calcium dependent proteins (mainly kinases) [73]. Release of intracellular Ca^2+^ is linked to apoptosis (programmed cell death) through the intrinsic pathway primarily involving processes that converge on caspase-3 signaling [87].

Moreover, Ca^2+^ signaling has a central role in triggering apoptosis in many kinds of cells [88]. Prolonged accumulation of mitochondrial Ca^2+^ may lead to a phenomenon known as the mitochondrial permeability transition (MPT), that is regarded by some researchers as a mechanism of pathological cell death, and by others as the regulation of apoptosis [88,89].

Describing calcium signaling, the role of calmodulin (CaM) must be mentioned. This ubiquitous 148-residue protein was shown to be a major Ca^2+^ sensor in non-muscle cells, that responds to and regulates intracellular calcium levels [90]. Upon binding of Ca^2+^ its conformation is altered and enabled to bind with target peptides or proteins [91]. CaM senses the changes in intracellular Ca^2+^, and tunes the activity of numerous protein kinases, such as CaMKs. One of them, CaMKIV, is expressed in distinct brain regions that regulate learning and memory, emotion, and motor function [92].

The complex of calmodulin and one of the calmodulin-dependent protein kinases (CaM-KK) activates calcium stimulation of insulin gene transcription, showing that Ca^2+^ ions can affect insulin synthesis in pancreatic beta-cells [93]. Furthermore, frequency- and intensity-modulated fluctuation in the cytosolic and organelles free Ca^2+^ concentration are transduced into signals able to control multiple molecular systems and cellular functions by the actions of other regulatory Ca^2+^-binding proteins. Nonetheless, CaM seems to be the most important calcium sensor in eukaryotic cells [94]. The role of CaM-dependent systems involved in cell migration, tumor cell invasiveness, and metastasis development has been discussed recently by Villalobo and Berchtold [94].

The total calcium balance is supported by the work of the calcium sensing receptor (CaSR), that modifies parathyroid hormone secretion or renal cation handling. In the parathyroid gland, the CaSR is located on the cell surface of chief cells and is composed of seven transmembrane domains. Calcium ions bind to CaSR, which allows this protein to monitor and regulate the amount of calcium in the blood. Vitamin D and calcium, both acting through negative regulation in the parathyroids, have been recognized for many years as key modifiers of parathyroid hormone (PTH) gene transcription, hormone synthesis, and parathyroid cell proliferation [95].

As briefly shown in this section, calcium represents a crucial signal for almost every aspect of cellular life, and because of that it is involved in complex signaling networks. However, even small disorders in calcium homeostasis can trigger destructive processes that contribute to a number of pathogenic states [73]. For this reason, calcium signaling remains an important component of many studies. When researchers develop the ability to trace calcium dynamics, novel targets and treatments of chronic human diseases can be developed [68,73].

## 4. Zinc

Zinc as a divalent metal ion plays an important role in numerous processes in the cells. Zn^2+^ is a multitasking tool required for the activity of many enzymes, regulating transcriptional processes, cell growth, and differentiation, as well as the immunological response. Such versatility of biological zinc functions suggests a huge cellular demand for this ion. After Iron, Zinc is the second most abundant transition metal within the human body [96]. The concentration of this metal differs among organs and tissues, and the brain is the most enriched location of this metal ion in the body [97]. The concentration of Zn^2+^ in this organ reaches levels 10 times higher than that observed in serum—this highlights the significant role of zinc signaling in the central nervous system (CNS) [98].

In physiological conditions, the majority of Zn^2+^ is bound by proteins and small chelators in both excitable and non-excitable cells. The remaining pool of free zinc is strictly controlled; this part of it is found to be released by the cell as a zinc signal. The zinc spark is a very interesting example of exocytosis of zinc ions observed in early development of activated mammalian egg cells [99,100]. The oocytes require zinc for proper maturation [101]. After fertilization, the cell releases accumulated zinc ions as a spark, and this phenomenon can be described by a set of parameters like amplitude and integrated intensity [102]. Previous studies performed on mouse cells indicate that a high amplitude zinc spark may be a useful biomarker that helps to select high quality embryos prepared for in vitro fertilization (IVF) procedure [102]. The zinc sparks are triggered by intracellular calcium fluxes [100]. The effect of such potent zinc releasing is correlated with the blocking of polyspermy soon after fertilization on the way of ovastacin (a zinc metalloendopeptidase) activity [103].

In nerve cells, zinc ions are co-released to the synaptic cleft with glutamic acid from zincergic neurons or zinc-enriched (ZEN) terminals located mostly in mossy fibers of the hippocampus [104,105]. Mossy fibers work as a specialized unit, the main task of which is to convert the signals it receives into the code necessary for memory formation in CA3 region of the hippocampus [106].

The release of zinc and its interaction with the receptors of the postsynaptic membrane plays a significant role in long-term potentiation (LTP) and thus contributes to synaptic plasticity, which has been largely studied using animal models [104,107,108,109]. Simultaneously, studies in rats have shown that chelatable zinc ion homeostasis, and their proportion outside and inside neurons, is disturbed during acute behavioral stress. This may lead to an increase in the influx of zinc ions into the hippocampal cells, which under these conditions surprisingly reduces the ability of mossy fibers to generate LTP [110].

The exact amount of free zinc ions in human presynaptic terminals is still intensively discussed, and the imaging of the true distribution of unbound Zn^2+^ in the brain and nervous system is still a major challenge for scientists. Some studies confirm that zinc is a locally acting neurotransmitter that passes through the synaptic cleft, interacting with receptors on the postsynaptic membrane [111]. However, the release of zinc ions into the synaptic cleft [96], or its neuromodulatory character, is still under discussion [112,113]. Nevertheless, there is experimental confirmation that zinc ions can be successfully released into the synaptic cleft as a result of electrical stimulation, for example [114]. Additionally, in some cases, enhancement of Zn^2+^-dependent potentiation is observed on the postsynaptic side, and may be regulated by changes in the concentration of other cations like K^+^ [108].

There are many indications that proper zinc management in zincergic neurons is related to the level of expression of various proteins, of which metallothionein 3 (MT-3) plays a significant role [115]. It is a specific isoform of metallothionein that is expressed in a significantly increased amount in nerve cells enriched in Zn^2+^ [115]. Therefore, this protein is recognized as a provider of zinc ions to the site of the formation of synaptic vesicles. Furthermore, due to the lack of saturation with metal ions under physiological conditions, MT-3 may be an element of the mechanism protecting the cell against the dangerous increase in the concentration of free zinc ions [116]. The transport of Zn^2+^ ions into the vesicle is possibly due to the activity of the ZnT-3 transporter [117]. It is worth mentioning that the same protein is associated with cadmium-dependent toxicity within the hippocampus [118].

**Figure 3:**
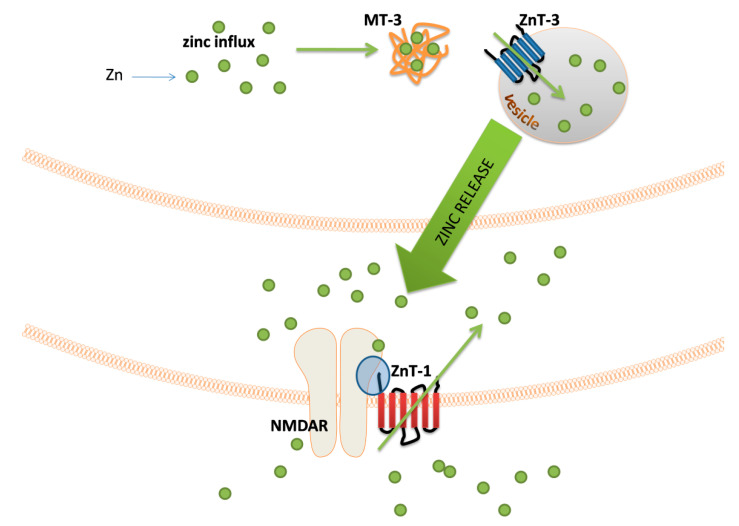
Schematic representation of the events related to the zinc signal release from zincergic synapses. After zinc influx, Zn^2+^ ions are bound by metallothionein 3 (MT-3). This protein delivers zinc to the zone where vesicles are formed. The influx of Zn^2+^ into the vesicle is catalyzed by ZnT-1 transporter. After release, Zn^2+^ appears in a synaptic cleft and diffuses through it. This phenomenon leads to the interaction of Zn^2+^ with postsynaptic N-methyl-D-aspartate glutamate receptor (NMDAR) and the influx of these ions into the postsynaptic nerve terminal. The level of Zn^2+^ in a postsynaptic neuron is regulated by the ZnT-1 transporter responsible for efflux of Zn^2+^ excess. The location of ZnT-1 in the membrane allows this transporter to interact with the GluN2A subunit of NMDAR (blue sphere).

In the membrane of postsynaptic neurons, zinc ions can cause a number of excitatory or inhibitory reactions by interacting with different receptors, and the best-known targets are N-methyl-D-aspartate glutamate receptor (NMDAR) [107,119], alpha-amino-3-hydroxy-5-methyl-4-isoxazolepropionic acid receptor (APMAR) [120,121], or voltage-gated Ca^2+^ channels [122,123]. The concentration of zinc ions in the postsynaptic nerve terminals is controlled by the ZnT-1 transporter activity; the expression of this transporter depends on the number of free zinc ions within the cell [124]. The ZnT-1 placement in the postsynaptic membrane closely correlates with the location of the NMDAR, and the transporter itself can interact with this receptor through the GluN2A subunit of the NMDAR [125]. A illustration of the sequence of events related to the zinc release by ZEN neurons is shown in Figure 3.

The influx of Zn^2+^ into the cell awakens the Ca^2+^ related processes, which is observed not only in neurons [126], but also in other cells [127].

The changes in concentration of free or loosely bound zinc with the aging of the organism cause disturbances in the functioning of various centers of the nervous system in old age [128]. The increased concentration of zinc ions in many areas of the brain is associated with the occurrence of serious diseases, such as neurodegenerative diseases, and the level of this metal concentration can be an indicator of disease progression [129]. Furthermore, the age-related changes in serum copper to zinc ratio may be used as an efficient biomarker of health disturbances, and the effect of the change on cellular processes in mostly non-excitable cells was very interestingly described in a review by Malavolta et al. [130].

Higher levels of free zinc have been detected in Parkinson’s disease (PD) [131] and Alzheimer’s disease (AD) patients [132] in the olfactory bulb. Moreover, intranasal zinc exposition causes the death of nasal cells by necrosis [133]. The specimens prepared from the autopsied brains of people with AD show a close correlation between the increased concentration of calcium and zinc ions and indicate that zinc ions may be involved in the dysfunction of neurons at the early stages of the disease development [129]. The dyshomeostasis and increase of unbound Zn^2+^ are dangerous for neurons and may lead to their damage and death [134]. One of the most supported mechanisms of neuronal death induced by free zinc excess was proposed to be a result of zinc accumulation within mitochondria [135]. It was suggested that this phenomenon leads to direct zinc-induced inhibition of cellular energy production, however, recent studies reveal that a more plausible mechanism relies on the dependence between calcium and zinc concentration [136,137]. Zinc ions directly participate only in the inhibition of mitochondrial movement, and calcium ions are responsible for mitochondrial damage [136].

Studies on the permeability of the blood-brain barrier (BBB) place the higher availability of zinc ions in a different perspective, and may suggest positive aspects of this phenomenon. The increased concentration of extracellular zinc can affect the capacity of the BBB. Zinc loosens the tight junction between endothelial cells of brain capillaries in it. This phenomenon facilitates the cleansing of the central nervous system of toxins and waste in pathological conditions such as ischemia, but it was also postulated as a part of normal brain function under physiological conditions [138].

The opposite phenomenon, zinc deficiency, can be equally harmful. Physiologically, the amount of zinc in the human brain decreases with age [139]. A decrease in the intracellular concentration of zinc ions is correlated with an increase in glutamate release, the activity of which induces apoptosis in neurons [140]. Furthermore, there are studies showing that zinc dietary deficiency leads to disturbances in cognitive functions [141]. The lowering of zinc concentration in the brain, mostly by poor dietary intake, is related to many pathological conditions, among which mental disorders are included [142]. Recent studies correlate depression with a low zinc level, and some of them treat this metal ion supplementation as a potential therapy for this serious mental condition [143].

## 5. Copper

Copper belongs to the transition metals group. This metal can exist in two different stable forms of ions in biological systems—Cu^+^ and Cu^2+^. This characteristic allows it to participate in redox reactions, which can have a negative effect if copper homeostasis is disturbed [144]. For this reason, it is required to keep the copper ions bound to proteins and to strictly control the concentration of free copper ions, both outside and inside the cell. Similarly to zinc, the highest concentration of copper has been detected in the brain [145]. Copper is very important for cell development and functions. This ion is involved in the defense strategy against free radicals by its presence in Cu/Zn superoxide dismutase (Cu/Zn-SOD). Disturbances in the folding of this protein are related to the development of serious dysfunction of motor neurons [146].

In the CNS, copper signaling may influence synaptic transmission indirectly by modulating the synthesis of neurotransmitters, e.g., via the peptidylglycine α-amidating monooxygenase (PAM) [147] and dopamine β-hydroxylase (DBH) [148] pathways, or directly by being released into the synaptic cleft from the terminals of glutamatergic neurons [149,150]. However, the signal associated with copper ions has a different effect depending on the region where it is released; in the amygdala, copper acts towards enhancement of LTP [151], while in the hippocampus it shows a tendency to inhibit synaptic plasticity [145]. Interestingly, the LTP inhibition induced by copper is located on the presynaptic side of the transmission [152].

The management of the intracellular pool of copper is supervised by a set of different proteins. Copper enters the cell, e.g., via the Cu transporter 1 (CTR-1) [153,154], and it is immediately captured in the cytosol by a group of chaperones that deliver these ions to their target sites. Such a rapid response to the intracellular increase in copper concentration is important due to its high redox reactivity. The chaperones transport this metal to the target location, such as mitochondria [155] or the area of secretory vesicle formation, whilst further copper release from the neuronal cell is possible through ATP7A, the major copper transporter in the brain [156]. After diffusion through the synaptic cleft, copper ions interact with NMDA and AMPA receptors and may also modulate the activity of γ-aminobutyric acid (GABA) and other amino acid receptors [157,158].

**Figure 4:**
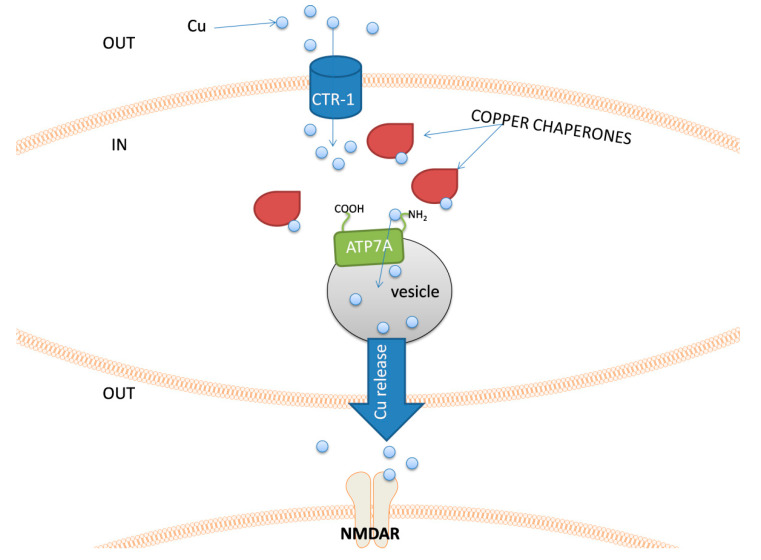
The schematic representation of an example sequence of events related to copper release possible in nerve cells. Copper ions are taken up to the cell by a Cu transporter 1 (CTR-1). The presence of free Cu^+^ ions immediately activates a set of copper chaperones. These proteins transport copper within the cell and deliver it to its destination. In neurons, that can release copper into the synaptic cleft, chaperones introduce copper ions to the ATP7A protein, which transport it to the lumen of a vesicle. After Cu release, it can interact with e.g., NMDAR.

An example of the cellular pathway related to the copper release by neurons is shown in Figure 4.

The natural aging of the brain is associated with copper accumulation [139]. The increased concentration of copper ions in the body is considered as a potential cause of cognitive decline observed in elderly people [159]. Interestingly, the results obtained after analyzing the brains of people suffering from AD showed the opposite phenomenon. In comparison to healthy patients, the concentration of copper was lower in the brain areas most affected by the action of this disease—hippocampus and amygdala [160]. Therefore, it has been suggested that such a significant reduction in the availability of copper ions may be an important factor in the pathogenesis of this disease [161]. This copper deficiency correlates with a simultaneous reduction of B12 vitamin availability, which altogether facilitates neurodegeneration [162].

Research shows that the action of copper ions may have a protective effect on nerve cells. On the molecular level, modulating the activity of the NMDA receptor through copper ions significantly reduces the influx of calcium ions, and thus reduces the risk of cell damage [163].

Disturbances in the expression of proteins related to the relocation of copper ions, such as ATP7A, lead to neuronal damage, mainly due to the hyperactivity of NMDA receptors. Therefore copper treatment of these neurons can reduce and reverse this phenomenon [163]. Additionally, the up-regulation of copper availability may be controlled by the iron regulatory protein 2 (IRP2), which can participate in the pathological redistribution of copper in neurodegenerative conditions [164].

The influence of copper ions on neurotransmission depends on their concentration and time of exposure, which can have an impact on normal brain function, and diseases such as AD and dementia. Studies on neuronal cell cultures revealed that acute exposure to Cu^2+^ ions increased the synaptic activity of neurons by the AMPA receptor pathway, but the changes provided by this phenomenon were reversed after 24 h of exposure, suggesting high activity of copper homeostatic mechanisms [165].

Simultaneously, animal studies have shown that chronic exposure to low doses of dietary copper led to an increase in its presence within the brain. This phenomenon directly induced greater neuronal degeneration and death following DNA damage and activation of apoptosis [166].

An important aspect of nerve cell function is access to glucose and its metabolism. It was shown that with aging, the amount of glucose in the brain increases, and its metabolism slows down [167]. Along with the fact that diabetes negatively affects copper ion transport mechanisms [168], researchers suggest that the highest level of glucose and reduced copper availability in the brain can serve as an indicator of neurodegeneration [167]. Such a relationship between the level of glucose metabolism and copper ion homeostasis can also be used in the adaptation of diagnostic techniques for the early detection of these dangerous changes by connecting positron emission tomography–computed tomography (PET/CT) diagnosis with ^64^Cu flux detection [169].

Apart from neurodegenerative diseases, mental disorders may also be caused by abnormal access to copper ions. In the case of socially isolated animals, a decrease in the concentration of copper ions in the brain has been detected and correlated with a reduction in cognitive functions and the development of depression [170].

## 6. Iron

Iron is an essential metal, necessary for practically all living organisms, present in the form of ferric and ferrous ion, i.e., Fe^3+^ and Fe^2+^. The ability to form two oxidation states is responsible for iron’s role in redox reactions. Iron overload could be as dangerous as its deficiency. As there is no regulatory mechanism of the removal of iron excess from the body, its absorption has to provide coverage of the requirements for iron ions, while at the same time preventing iron overload. Therefore, perfect balance between iron absorption, distribution, accumulation, and excretion should be maintained. Iron sensing and signaling are mostly related with its homeostasis in the human body, in which several mechanisms are involved.

Dietary iron is absorbed in enterocytes in the small intestine, both as heme and non-heme iron. Heme-Fe is absorbed more efficiently than the non-heme one, likely due to the lack of essential interactions with dietary factors in the gastrointestinal tract [171]. Heme carrier protein (HCP1) was found to mediate the transport of heme-Fe to the enterocytes, but it was lately identified as folate transporter [172,173]. Nevertheless, the study of Le Blanc et al. showed, that HCP1 is involved in low-affinity heme-Fe transport [174]. Inside the enterocyte, ferrous ion from heme is released in the process mediated by heme oxygenase (HO) and enters the same pathway of utilization as non-heme iron [171]. Fe^3+^ must be reduced to Fe^2+^ before it can be transported by the divalent metal transporter 1 (DMT1) inside the enterocyte. Such reduction is made by duodenal cytochrome B (Dcytb), located in the apical membrane of enterocytes, other ferrireductases, or by non-enzymatic reductants such as ascorbate and/or superoxide, probably also amino acids, e.g., cysteine [175]. Inside the enterocyte, up to 4500 iron ions can be stored in the complex with ferritin, a spherical protein consisting of 24 subunits of light and heavy type subunits [176]. Heavy chains, responsible for the reuse of iron ions, present ferroxidase activity, and reduction to Fe^2+^ allows the mobilization of these ions, and their subsequent use or efflux via the basolateral membrane of the enterocyte.

The export of iron is provided by ferroportin-1 (FPN1, SLC40A1). FPN1 is associated with hephaestin, ferroxidase which oxidizes exported Fe^2+^ again to Fe^3+^, the Fe^3+^ is bound to transferrin (Tf) in the blood, and then transported to the cells [175]. Apo-Tf possess two iron-binding sites, and to be recognized by the transferrin receptor (TFRC, TFR1) both sites must be occupied. One TFRC is able to bind two holo-Tfs, and such complex is transported through the cell membrane and located in endosome. Inside the endosome the environment is acidified, Fe^3+^ is reduced to Fe^2+^ by STEAP3 protein, and then exported to the cytosol by DMT1. Occurring simultaneously with the iron ion reduction, the Tf-TFRC complex hydrolyzes, TFRC is recycled to the cell membrane, and apo-Tf is released outside the cell [177].

The regulation of proteins involved in iron metabolism takes place by the iron-sensing mechanism during post-transcriptional modification. Iron responsive elements (IREs) are hairpin structure fragments of mRNA located at 5′-untraslated region (5′-UTR) or 3′-UTR. One or two types of iron-responsive elements binding proteins (IRE-BP) can be bound to the IRE, namely iron regulatory protein 1 (IRP1) and IRP2. When iron is scarce, the IRE-BP binds to IRE with high affinity, which results in suppression of translation for mRNAs with IRE located on 5′-UTR (e.g., ferritin, both light and heavy chains, or FPN1). When IRE is located in the 3′-UTR, binding of IRE-BP enhances the mRNA stability, and encoded protein is synthesized (e.g., TFRC and DMT1). That allows the import of iron ions into the cell. When the desirable iron level is achieved, the labile iron pool (LIP) increases, and Fe^2+^ from LIP can bind to IRE-BP causing conformational changes, resulting in detachment of the Fe-IRE-BP complex from IRE. Translation of proteins encoded by mRNAs with IRE in 5′-UTR becomes possible (e.g., iron storage protein ferritin or exporting FPN1), while mRNA with IRE in 3′-UTR becomes sensitive for nuclease attack and undergoes degradation [175,176].

Besides the regulation of uptake by the cell, iron absorption needs to be controlled in the gastrointestinal tract. Iron-dependent regulation of this metal absorption from enterocytes is provided by the peptide hormone, hepcidin, which was shown to be the key regulator of iron homeostasis. This 27 kDa peptide is expressed in hepatocytes, in Kupffer cells, and also in small quantities in macrophages and adipocytes [178,179]. It is encoded by the hepcidin antimicrobial peptide (HAMP) gene, mutations of which are connected with severe iron overload diseases and hemochromatosis. Hepcidin is produced as pre-pro-peptide composed of 84 amino acids, which is processed to the 60 amino acid pro-hepcidin, and then to the 25 amino acid active form [179,180]. Hepcidin binds to FPN1 which triggers its degradation, resulting in iron sequestration inside cells (such as enterocytes, hepatocytes or macrophages). This leads to a decrease in the amount of iron available for erythropoiesis, what in turn causes reduction in the level of hepcidin. As a result, iron absorption in the gastrointestinal tract increases, along with ion release from iron stores [176].

The excretion of hepcidin is regulated by several factors, including iron-dependent proteins: human homeostatic iron regulator protein (HFE, hemochromatosis protein), Transferrin Receptor 2 (TFR2), Hemojuvelin (HJV), and Transmembrane Serine Protease 6 (matriptase-2, TMPRSS6). All of these proteins are expressed in the liver. Liver iron content and serum iron (bound to Tf) affect hepcidin expression in a different manner [181].

**Figure 5:**
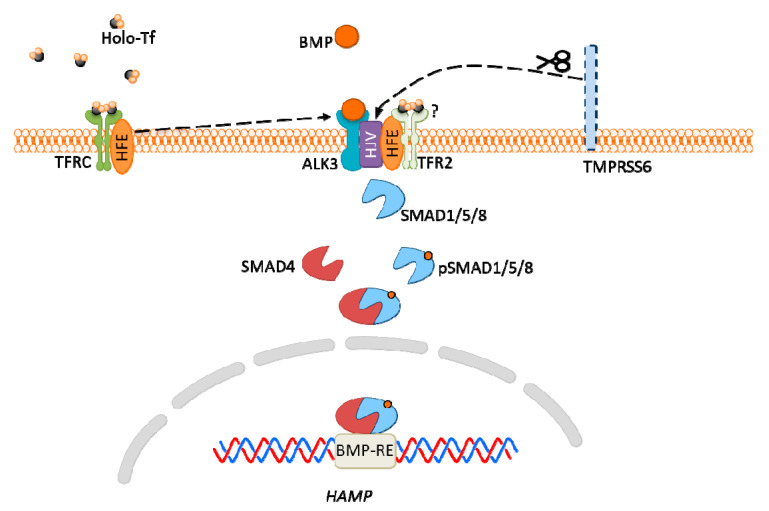
Hepcidin expression via bone morphogenic protein (BMP)-SMAD signaling pathway in hepatocytes. High iron concentration in plasma increases transferrin saturation, which results in homeostatic iron regulator protein (HFE) detachment from the transferrin receptor (TFRC)/HFE complex, then its interaction with ALK3 (BMP receptor), which leads to increased expression of ALK3 and activation of the BMP-SMAD signaling pathway. High plasma iron also causes stabilization of TFR2, which is associated with HFE, and may also interact with ALK3 (but this is not confirmed). Hemojuvelin (HJV) is a co-receptor for BMP, and TMPRSS6 protein cleaves HJV acting as an inhibitor of HJV expression.

The main regulator of hepcidin expression is bone morphogenic protein (BMP)-SMAD signaling pathway. In general, BMP triggers the phosphorylation of SMAD1/5/8 proteins, which subsequently bind to SMAD4 and translocate to the nucleus, where the complex activates hepcidin expression via interaction with BMP responsive element (BMP-RE) in the HAMP gene (Figure 5) [182].

HFE interacts with TFRC, at the binding site for holo-Tf [183]. Therefore, when serum iron-Tf concentration is high, HFE detaches from TFRC and likely interacts with active receptor-like kinase 3 (ALK3), the BMP receptor. The formed complex prevents ALK3 degradation, increases its expression, which results in hepcidin expression [184].

The TFR2 protein also functions as an iron sensor. Under conditions of high iron concentration, it is stabilized by binding iron-Tf and is associated with HFE; however, it is unclear if TFR2 also interacts with ALK3. *Tfr2* knockout mice exhibited decreased BMP-SMAD signaling and hepcidin expression. The effect is even more visible in double-knockout *Hfe*/*Tfr2* mice [182,185].

HJV was found to be a co-receptor for BMP, as mutations in *Hjv* gene results in hemochromatosis, a disease manifested by iron overload. Interestingly, *Hjv^−/−^* mice exhibited both hepcidin expression and the BMP-SMAD signaling pathway, yet they were significantly attenuated. This results suggest that HJV may act not as a direct sensor, but as an enhancer of iron signaling in the hepcidin pathway [186].

TMPRSS6 protein cleaves HJV, therefore it acts as an inhibitor of HJV expression. The deficiency of TMPRSS6 causes unregulated BMP signaling, the hepcidin excess and anemia resulting from iron deficiency [187].

Studies on transgenic and wild type mice showed that higher amounts of iron in the diet result in higher hepatic iron levels, along with increased amounts of mRNA of enzymes responsible for ROS inactivation (*Sod1* and *Sod2*) [188]. Moreover, hepcidin levels reach a plateau with a certain supply of iron. Further increases in the amount of iron in the diet results in the rise of iron concentration in the liver, what does not affect hepcidin regulation [188]. This result indicates that the knowledge of iron regulation via its concentration in the liver needs to be expanded.

The iron exporter, FPN1, is composed of 571 amino acids. Based on data from the model and the crystal structure of a putative bacterial homologue, it has 12 helical transmembrane domains, with the C- and N-terminus positioned intracellularly [189,190]. Mutations in the *SLC40A1* gene lead to hemochromatosis type 4 (HC4), also called ferroportin disease [191,192].

Iron is essential for the development of all vital organs, including the brain. Iron deficiency in infancy results in the development of cognitive, motor, socio-emotional, and neurophysiological disorders [193]. Therefore, significant amounts of iron ions are absorbed in duodenal enterocytes, despite the fact that breast milk contains relatively little iron. During suckling more than 80% of iron is absorbed, while shortly after weaning the percentage decreases to 10–20%. The reason for such changes is not clear. It was shown that in the immature digestive system, the process of iron absorption is hypo-responsive to the inhibitory effect of hepcidin [194]. Nevertheless, ferroportin was shown to be pivotal for high iron absorption, as it was significantly decreased in ferroportin knockout mice.

Elevated iron levels were found in tumors, as they exhibit increased metabolism and rapid proliferation of the cells [195,196,197]. Moreover, increased iron dietary uptake and/or systemic iron levels correlate with an increased risk of developing certain types of cancer, including colorectal cancer (CRC) [198]. It was shown that iron demand is a key factor during CRC development, but its character varies in different CRC cell types [199]. Xue et al. presented results of investigations showing that elevated iron concentration in the cell leads to an activation of the signaling pathway involving Cyclin dependent kinase 1 (CDK1), Janus kinase 1 (JAK1) and Signal transducer and activator of transcription (STAT3), i.e. CDK1-JAK1-STAT3 signaling pathway [200]. This results in tumor cell proliferation, and may partially explain how high iron diet intake increases the risk of CRC development.

In 2012, a new form of non-apoptotic programmed cell death was identified. Morphological and biochemical characteristics of ferroptosis, induced by iron-dependent lipid peroxidation, include ROS accumulation (from iron metabolism), NADPH oxidase activity and lipid peroxidation products, cell volume shrinkage, and increased mitochondrial membrane density, with lack of typical apoptotic and necrotic features [12,201]. It was shown, that after the induction of ferroptosis by erastin, the cell death can be inhibited by iron chelators, such as deferoxamine, and antioxidant vitamin E, which provides evidence that ferroptosis depends on iron and ROS production [202]. The details of the dependence of ferroptosis on iron still need to be explained. In addition to iron chelators, ferroptosis can be inhibited by energy stress, while AMP-activated protein kinase (AMPK), the energy sensor in cell, is activated [203]. This promotes poly-unsaturated fatty acids (PUFAs) and other fatty acids biosynthesis, what results in ferroptosis inhibition.

Besides ferroptosis, iron overload and ROS generation can induce apoptotic cell death [204]. Recent results suggest that in myelodysplastic syndrome (MDS) patients, iron overload was related to the decrease of hypoxia inducible factor 1-a (HIF1-a). This process was shown to be iron concentration dependent as Fe^2+^ is a cofactor for prolyl hydroxylase domain 2, (PHD2), responsible for HIF1-a degradation. Triggering the HIF1-a/ROS signaling pathway led to mitochondrial-dependent apoptosis. On the other hand, apoptosis induced by H_2_O_2_ in colon-adenocarcinoma cell line (Caco-2) was abrogated by zinc or iron chelator, bathophenanthroline disulfonic acid (BPDS). Simultaneously with cell death induction, RNA binding activity of IRP1 increased. It resulted in increased DMT1 expression and iron uptake. Inhibition of this process by zinc means that zinc can act as an effective modulator of H_2_O_2_-induced iron signaling and cell death [205].

Additionally, superparamagnetic iron oxide nanoparticles (SPIONs), used as MRI contrast agents, were found to initiate autophagy processes in macrophages, in a manner suggesting that macrophages recognize SPIONs as microorganisms and their removal is carried out in a similar fashion [206]. Furthermore, SPIONs induced autophagy by increasing p62 mRNA expression, resulting from toll-like receptor 4 (TLR4) activation. Moreover, iron oxide nanoparticles (IONPs) have been found to reduce the expression of osteoclastogenesis-related genes. The way of action of IONPs is based on the inhibition of osteoclastogenesis through regulating the signaling complex involving the p62 protein (TRAF6-p62-CYLD) also [207].

## 7. Summary and Conclusions

Living cells simultaneously use various intracellular signaling systems to sense and interpret changes in their extracellular environment.

Many controversies still exist regarding the roles of magnesium in cell signaling. These controversies are caused mainly by the versatility and complexity of this metal ion. Studies show that it can be trophic or toxic, an activator or an inhibitor, and a cause of disease progression or regression. Magnesium has a crucial role in numerous cellular processes including enzymatic reactions, ion channel functions, metabolic cycles, and DNA/RNA stabilities. Disorders of Mg^2+^ homeostasis are involved in neurodegenerative and cardiovascular diseases, bone disorders, asthma, cancer, and diabetes. The hydrated magnesium cation is hard to dehydrate, which makes Mg^2+^ionsvery difficult to pass through narrow channels in biological membranes. Although a conserved magnesium-binding protein has not been reported for Mg^2+^ signaling pathways, phosphoryl transfer reactions in cells depend on this ion. Moreover, Mg^2+^ ions, because of their abundance and multivalence, are main players in neutralizing negatively charged biomolecules such as ROS or nucleic acids. Calcium cations should be better competitors for binding to negatively charged biomolecules, however, under normal and resting conditions the intracellular calcium ions are maintained at low levels. Calcium is a universal second messenger used to regulate versatile cellular processes ranging from contraction, through proliferation, secretion, fertilization, to learning and memory. Moreover, calcium signaling is essential for T cell activation, tolerance of self-antigens, differentiation, and development. Cells acquire signal Ca^2+^ ions from both internal and external sources. Calcium signals are detected and transmitted to downstream responses by a set of Ca^2+^ binding proteins that function as calcium sensors, with the prevailing role of the calmodulin protein. In normal conditions, free calcium concentration [Ca^2+^] is maintained at approximately 100 nM; much greater levels are stored in the ER and mitochondria. In electrically non-excitable cells, the Ca^2+^ ions are released from internal stores mainly in a process known as the store-operated calcium entry (SOCE). This mechanism has two important functions in the cell: cellular signaling and store refilling. Ca^2+^ flux through the excitable cell membrane is strictly limited in time due to biophysical properties of VGCCs and ligand-activated receptors. During calcium-mediated signal transduction these receptors and channels associate temporally and form the transient signaling complexes.

Zinc and transition metal ions, such as copper and iron, have been traditionally thought to be kinetically inert cofactors that are bound and buried within proteins and other molecules. However, recent data reveal that beyond their traditional functions as metabolic cofactors, they have also an important role in cell signaling. Copper, iron, and zinc are the three most abundant non-alkali metal ions in the brain, for this reason both deficiency and excess of these metal ions results in central nervous system disease.

Zinc might regulate the plasticity of synapses, however, how much zinc is released during synaptic activity remains highly elusive. Some research confirms that Zn^2+^ ions facilitate the transduction of a variety of signaling cascades in response to extracellular stimuli. Zinc pathways interact with calcium, redox, and magnesium signaling. The influx of Zn^2+^ into the cell triggers the Ca^2+^ related processes, what was observed not only in neurons but also in other cells. In physiological conditions, the majority of Zn^2+^ is bound by proteins and small chelators in both excitable and non-excitable cells. Zinc has an impact on the activity of many enzymes, transcriptional processes, cell growth and differentiation, and the immunological response. Very interesting events caused by zinc ions are called “zinc sparks”; functionally, the zinc sparks mediate a decrease in intracellular zinc content that is necessary for continued egg cell cycle progression. Recent studies reveal that this mechanism is dependent on intracellular calcium transients, which are tightly associated with embryonic development.

Copper ions may exist in two different forms in biological systems—Cu^+^ and Cu^2+^. Because of their potential toxicity to cause oxidative stress and free-radical damage, copper ions must be strictly controlled, e.g., through binding to specific proteins. The exchange of copper between a variety of target-specific cytosolic chaperones and their targets is driven presumably by an increase in the copper binding affinity. Copper containing enzymes and transcription factors are essential for cellular integrity, energy production, and proliferation. The intracellular level of copper is managed by a set of different proteins such as Cu-transporters and copper chaperones. Copper signaling may influence synaptic transmission indirectly by modulating the synthesis of neurotransmitters, or directly by being released into the synaptic cleft from the nerve terminals of glutamatergic neurons.

Iron is present in the living cells in the form of ferric and ferrous ion, i.e., Fe^3+^ and Fe^2+^. There is no regulatory mechanism for the removal of excess iron from the body. Therefore, the perfect balance between iron absorption, distribution, accumulation, and excretion should be maintained. Regulation of the proteins involved in iron metabolism is a form of iron-sensing mechanism during post-transcriptional modification. Elevated iron levels were found in tumors, as they exhibit increased metabolism and rapid proliferation of the cells. Iron ions, like copper ions, take part in the redox signaling process. The distinction between signaling and toxic redox processes is not always obvious. There is growing evidence that iron excess is a major risk for carcinogenesis, suggesting the importance of ferroptosis-resistance. Ferroptosis is a relatively recently described type of non-apoptotic cell death, induced by iron-dependent lipid peroxidation, that remains to be fully characterized.

There are suggestions that copper and iron redox mechanisms are interesting therapeutic targets for treating some diseases such as cancer and chronic lung inflammation.

Table 1 summarizes transporters and receptors engaged in the signaling of the metal ions characterized in this review, as well as their downstream signaling events.

This review shows the role of chosen metal ions in signaling processes. As we can see, variable thermodynamics and kinetics of metal actions blur the lines between metabolism and signaling, placing metals in a unique chemical and biological space.

Nonetheless, deficiency, as well as excess, of any essential metal ion, can lead to many disorders. Not only redox-inactive alkali metals, but also some transition metal ions, have important roles in signaling and other biological processes. Recent studies focus on the disruption of transition and non-transition metal ions homeostasis by such factors as the disturbances of their bioavailability, and changes in the chemical status of essential cations. This knowledge can help to prevent many disorders, and to design proper medication, such as inhibitors of the destructive signaling pathways, where these metal ions play an important role.

Many conclusions on the distribution of metal ions in excitable cells are based on conventional histochemical studies of nerve tissues. This technique is used primarily to show sites of only relatively high metal accumulation. Hence, the main far-reaching goal of novel research seems to be the improvement and development of techniques able to detect the chosen chemical form, concentration, and location of metal ions, especially zinc, iron, and copper, e.g., in the living brain. As described in this review, the proper cellular management of the copper and zinc levels is relevant for signal transduction and viability of neuronal cells. Additionally, the potent regulation of copper and zinc signals in excitable cells strongly influences calcium concentration.

## Figures and Tables

**Table 1:** Summary of described signaling functions of chosen metal ions.

Metal	Metal Ion Location during Signal Induction	Transporters and Receptors Involved in Sensing	Downstream Results
Mg	Intracellular	HIP14 MRS2 MMgT1/2	- suppression of ROS toxicity - dynamics of cytoskeleton - ribosomal biosynthesis, regulation of translation via mTOR pathway, - antagonizing the Ca^2+^ signal - PTP inhibition - repair of DNA damage - inhibition of protein aggregation
	Extracellular	TRPM6 and TRPM7 MagT1 NIPA SLC41A1(controversial: Na^+^/Mg^2+^ transporter or Mg^2+^ sensor) CNNM (controversial: Na^+^/Mg^2+^ transporter or Mg^2+^ sensor)	- growth factor signaling - membrane stabilization - channel regulation - osteoblast apoptosis
Ca	Intracellular	IP_3_Rs RYRs NFAT NAADP cADPR Calmodulin STIM1	- gene transcription - T-cell activation and development - CaMKs activation - insulin synthesis - fertilization - learning and memory
	Extracellular	TRPMs TRPCs VGCCs Kinases Caspase-3 CaSR Orai1 (Calcium Release-Activated Calcium Modulator 1)	- membrane potential modulation - response to many kinds of stresses - signal transduction - neuronal synaptic transmission - apoptosis - regulation of PTH - cell proliferation, mobility
Zn	Intracellular	MT-3 ZnT-3 ZnT-1	- modulation of mitochondrial activity - vesicle formation - regulation of postsynaptic signal transduction
	Extracellular	NMDAR APMAR VGCCs	- synaptic signal transduction and/or neuromodulation
Cu	Intracellular	Chaperones ATP7A	- proper functioning of mitochondria - vesicle formation - regulation of cell redox processes
	Extracellular	PAM DBH NMDAR AMPAR	- neurotransmitter synthesis modulation - reduced influx of Ca^2+^ - modulation of GABA and other amino-acids receptors
Fe	Intracellular (LIP)	RE-BP bound to IRE at mRNA CDK1-JAK1-STAT3 pathway PHD2	- ferritin and FPN1 translation; suppression of translation of TFRC and DMT1 - tumor cell proliferation - apoptosis - ferroptosis
	Extracellular (iron-Tf)	HFE/TFRC TFR2	- hepcidin expression
